# Lymphatic filariasis baseline survey in two sentinel sites of Ogun state, Nigeria

**DOI:** 10.11604/pamj.2015.20.397.5686

**Published:** 2015-04-23

**Authors:** Patricia Nkem Okorie, Emmanuel Davies, Olushola Omoniyi Ogunmola, Olusola Ojurongbe, Yisa Saka, Bridget Okoeguale, Ekanem Ikpi Braide

**Affiliations:** 1 Institute for Advanced Medical Research and Training, College of Medicine, University of Ibadan, Ibadan, Nigeria; 2 Ministry of Health, Nigeria; 3 Department of Medical Microbiology and Parasitology, Ladoke Akintola University of Technology, Osogbo, Osun State, Nigeria; 4 Department of Zoology, Federal University Lafia, Nasarawa State, Nigeria

**Keywords:** Lymphatic filariasis, Mosquitoes, Anopheles gambiae s.l, Culex species, Wucheraria bancrofti, Nigeria

## Abstract

**Introduction:**

In preparation for Mass Drug Administration by National Lymphatic Filariasis Elimination Programme, a baseline epidemiological investigation on lymphatic filariasis (LF) was conducted in two sentinel sites of Ogun State, Nigeria. The study was carried out in Ado-Odo Ota and Abeokuta South Local Government Areas (LGAs) to determine LF prevalence, microfilarial density and the abundance of Wucheraria bancrofti in the mosquito vectors.

**Methods:**

Microscopic examination of thick blood smears of 299 and 288 participants from Ado-Odo Ota and Abeokuta South LGAs was conducted. Visual observations of clinical manifestations of chronic infection and questionnaire administration were also conducted. Indoor resting mosquitoes were collected using the pyrethrum spray technique and CDC light traps and mosquitoes were dissected for filarial larvae.

**Results:**

Microfilaria prevalences were 4.0% and 2.4% in Ado-odo Ota and Abeokuta South LGAs. The microflarial density (mfd) was 30.6mf/ml and 23.9 mf/ml in the same areas. No clinical manifestations of the infection were found at both sites. Knowledge of LF by inhabitants was very low in the two areas. Anopheles gambiae s.l and Culex species mosquitoes were collected but none was found positive for stage L3 infective larvae.

**Conclusion:**

Mass awareness campaigns on the goal of mass drug administration, cause of LF, mode of transmission, the relationship between infection and clinical signs/symptoms is advocated so as to increase acceptance and support of the control programme by the community.

## Introduction

Lymphatic filariasis (LF) is a neglected tropical disease (NTDs) caused by parasitic worms and transmitted to humans by mosquitoes [1]. In Africa, it is caused by Wuchereria bancrofti and transmitted by Anopheles and Culex mosquitoes [1, 2]. An estimated 1.34 billion people in 81 countries are at risk of infection by the disease and about 40 million people suffer from the clinical manifestations of the disease [1, 2]. This includes 15 million with lymphoedema (elephantiasis) and 25 million men who have urogenital swelling, especially scrotal hydrocele [1]. About 30% of people at risk reside in the African region while 65% of those at risk reside in South-East Asia Region, and the remainder in other parts of the world [1]. The Global Program to Eliminate Lymphatic Filariasis (GPELF) was launched in the year 2000 with the aim of interrupting transmission and reducing morbidity and preventing disability [1]. Interruption of transmission is possible through mass drug administration (MDA), using once-yearly treatment with a single dose of albendazole plus either ivermectin or diethylcarbamazine (DEC) for 4-6 years [1]. WHO guideline for monitoring and evaluating coverage and impact of LF control programmes is that the sentinel site should be used to ascertain that the baseline indicators will make it feasible to conduct periodic evaluation of the parasitological indicators during MDA [1]. Nigeria has the highest number of cases of LF in Africa and ranks third globally [3]. About 106 million people in Nigeria are at risk of the disease [4]. The main vectors of LF in Nigeria are mosquitoes of the An. gambiae (principally An. gambiae s.s. and An. arabiensis) and Anopheles funestus complexes [5–7]. Lymphatic filariasis is prevalent in all states and geopolitical zones of Nigeria and a total of 241 lymphodema and 205 hydrocele cases have been reported from mapping surveys conducted in the country [4, 7].

Nigeria started implementing MDA in 2000 in 4 LGAs (Wamba & Akwanga in Nasarawa State and Kanke & Pankshin LGAs in Plateau State) and expanded to 195 LGAs in 2012. Between 2000 and 2012, MDA has been administered in 19,915,586 million people [8]. Significant progress has been made in the use of MDA for LF control in Nigeria and Only 13 LGAs in Borno State is yet to be mapped. In Ogun state, LF is endemic in 14 out of the 20 local government areas (LGAs). In order to achieve the goal of LF elimination by 2020, National LF elimination program of Nigeria needs to scale up MDA in all the states of the federation. In preparation for MDA in Ogun State, epidemiological mapping was conducted in 15 LGAs in 2003 and 5 LGAs in 2011 by the national LF elimination programme (NLFEP) using immunochromatographic card test (ICT). Mapping results reported a prevalence of 1-4% and 4-10% in 2003 and 2011 respectively [4]. Mapping results from Abeokuta South and Ado odo Ota LGAs reported a prevalence of 4 and 10% respectively [4]. Based on the mapping results, the implementation units (LGAs) for LF MDA were determined. This study presents the baseline data on the distribution and the level of infection of LF in two LGAs in Ogun State which will form the base for the implementation of MDA in all endemic LGAs in two Senatorial Districts (i.e. Ogun Central and Ogun West) of the state.

## Methods

**Study areas:** the study was carried out in (1) Ijeun Lukosi, an urban community in Abeokuta South, Ogun Central Senatorial District and (2) Alapoti, a rural community in Ado-Odo Ota Local Government Area, Ogun West Senatorial District of Ogun State where mapping with ICT has previously been conducted in 2003 and 2011 respectively. Abeokuta south LGA is located between 7°09′00′′N and 3°21′00′′E and has an area of 71 km^2^ and a population of 250,278 at the 2006 census. Ado Odo Ota LGA is located at 6°41′00′′N 3°41′00′′E and has an area of 878 km^2^ and a population of 526,565 at the 2006 census. The study was conducted in September 2013.

**Census and mapping:** prior to commencement of the study, the aim of the study was explained to the village and the members of the community were sensitized by door to door visits of each household. A rally was then held were all consenting individuals aged from 5 years and above participated in the study. This study was approved by Nigeria Federal Ministry of Health as one of its programmatic activities.

**Parasitological survey:** blood sampling for parasitological examination took place in the night between 22:00 and 02:00. A thick blood film was prepared from finger -prick blood drawn into a into a blood collection tube (60 µL) and then stained with Giemsa. Microscopic examination of slides was done. Microfilariae were identified based on the specific morphological features [9]. The prevalence of microfilaraemia was determined and the intensity of infection was expressed as mf/ml [10].

**Clinical examination and questionnaire administration:** each participant was examined by trained health workers for symptoms and signs of lymphatic filariasis. A structured questionnaire was administered to obtain information on the subjects’ demographic data (age, sex, education, occupation, e.t.c), knowledge of the cause and control of the disease.

**Mosquito Collection**. Indoor resting mosquitoes were collected using the pyrethrum spray technique and CDC light traps. Mosquito collection was carried out in at least 20 houses in each of the LGA for 2 days each. Mosquitoes were identified using standard morphological keys and categorized according to the species [11, 12]. Each mosquito was dissected under a binocular dissecting microscope and observed for the presence or absence of the filarial larval stages (L1-3).

**Data analysis:** the microfilaria prevalence per site was calculated as the proportion of blood slides found positive for microfilariae. The microfilarial density (mfd) per site was calculated as the average number of microfilariae in slides found positive for microfilariae per ml of blood (presuming 60 µl per slide) [10]. Data was analysed using STATA software (version 12, StataCorp, Texas, USA). Proportions were compared using Chi Square (χ^2^) tests and differences in the intensity of infection was tested using student t test (two sided) at P = 0.05.

## Results

A total number of 443 individuals from Ado-Odo Ota were mobilized for blood collection. Blood samples were successfully collected from 299 individuals (156 females and 143 males) within the specified period. In Ado-Odo Ota, more than forty percent (44.8%) were students while 23.7% and 12% were traders and farmers respectively (Table 1). More than seventy percent (72.2%) were aged from 5 to 49 years and the age group with the highest number was age group 10-19 (30.8%) (Table 2). Out of the 299 individuals examined 12 were positive by microscopy giving an overall prevalence of 4% (Table 2). The prevalence among females 4.5% was significantly higher than in males (3.5%) (χ2 = 12.6357, p = 0.0000). The younger age group (5-49 years) had significantly higher infection than the older age groups (50+ years) (χ2 = 3.9185, p = 0.048). The prevalence infection was distributed among the different age groups from 5 to 79 years, except for age group 30 to 39 where no infection was recorded (Table 2). The microfilarial density (mfd) of the 12 positive individuals was 30.6mf/ml. No significance difference was observed between the microfilarial density of males (36.7 mf/ml) and females (26.2mf/ml) (t = 1.0035, p = 0.3306) (Table 2). A total number of 369 individuals from Abeokuta South LGA were mobilized for blood collection. Blood samples were successfully collected from 288 individuals (175 females and 113 males) within the specified period. In Abeokuta, 53.5% and 25% of the participants were students and traders respectively (Table 1). More than eighty percent (85.4%) were aged from 5 to 49 years and the age group with the highest number was age group 10-19 (33.8%) (Table 3). Out of the 288 individuals examined 7 were positive by microscopy giving an overall prevalence of 2.4% (Table 3). There was no significance difference between the prevalence in females 2.9% and in males (1.8%) (χ2 = 0.3423, p = 0.559). Prevalence of infection was recorded in age groups 10 to 19, 30 to 39 and 40 to 49 only. The microfilarial density (mfd) of the 7 positive individuals was 23.9mf/ml (Table 3). No significance difference was observed between the microfilarial density of males (25.1mf/ml) and females (23.9mf/ml) (t = 1.3646, p = 0.7201). Also, there was no significant difference in the overall microfilarial density (mfd) of Ado-Odo Ota and Abeokuta South (t = 0.9779, p = 0.3427).

**Table 1:** Occupation of participants in Ado-Odo Ota and Abeokuta South LGA, Ogun state

Occupation	Ado-Odo Ota		Abeokuta South	
	No.	%	No.	%
Artisan	24	8.0	26	9.0
Civil servant	4	1.3	8	2.8
Driving	8	2.7	7	2.4
Farming	36	12.0	3	1.0
Retiree	3	1.0	4	1.4
Student	134	44.8	154	53.5
Teaching	5	1.7	2	0.7
Trading	71	23.7	72	25.0
Clergy	0	0.0	5	1.7
Nursing	1	0.3	0	0.0
Others	13	4.3	7	2.4
**Total**	**299**	**100**	**288**	**100**

**Table 2:** Prevalence of lymphatic filariasis in Ado-Odo Ota LGA, Ogun state

Age	Total			Female			Male		
	No. examined	No. positive (%)	Total mfd (mf/ml)	No. examined	No. Female		No. examined	No. Male	
					positive (%)	mfd (mf/ml)		positive (%)	mfd (mf/ml)
5 to 9	38	1(2.6)	16.7	20	1(5.0)	16.7	18	0(0)	0.0
10 to 19	92	2(2.2)	50.1	50	1(2.0)	16.7	42	1(2.4)	83.5
20 to 29	44	2(4.5)	25.1	16	0(0)	0.0	28	2(7.1)	25.1
30 to 39	42	0(0)	0.0	23	0(0)	0.0	19	0(0)	0.0
40 to 49	37	3(8.1)	22.3	22	3(13.6)	22.3	15	0(0)	0.0
50 to 59	22	1(4.5)	33.4	9	0(0)	0.0	13	1(7.7)	33.4
60 to 69	12	2(16.7)	41.8	8	2(25)	41.8	4	0(0)	0.0
70 to 79	6	1(16.7)	16.7	4	0(0)	0.0	2	1(50.0)	16.7
80 to 89	1	0(0)	0.0	0	0(0)	0.0	1	0(0)	0.0
unknown	5	0(0)	0.0	4	0(0)	0.0	1	0(0)	0.0
**Total**	299	12(4.0)	30.6	156	7(4.5)	26.2	143	5(3.5)	36.7

**Table 3:** Prevalence of lymphatic filariasis in Abeokuta south LGA, Ogun state

Age	Total			Female			Male		
	No. examined	No. Total		No. examined	No. Female		No. examined	No. Male	
		positive (%)	mfd (mf/ml)		positive (%)	mfd (mf/ml)		positive (%)	mfd (mf/ml)
5 to 9	54	0(0)	0.0	27	0(0)	0.0	27	0(0)	0.0
10 to 19	97	4(4.1)	25.1	57	2(3.5)	25.1	40	2(5)	25.1
20 to 29	41	0(0)	0.0	28	0(0)	0.0	13	0(0)	0.0
30 to 39	29	2(6.9)	25.1	19	2(10.5)	25.1	10	0(0)	0.0
40 to 49	25	1(4)	16.7	19	1(5.3)	16.7	6	0(0)	0.0
50 to 59	19	0(0)	0.0	13	0(0)	0.0	6	0(0)	0.0
60 to 69	12	0(0)	0.0	10	0(0)	0.0	2	0(0)	0.0
70 to 79	10	0(0)	0.0	2	0(0)	0.0	8	0(0)	0.0
80 to 89	1	0(0)	0.0	0	0(0)	0.0	1	0(0)	0.0
**Total**	**288**	**7(2.4)**	**23.9**	**175**	**5(2.9)**	**23.4**	**113**	**2(1.8)**	**25.1**

**Clinical manifestation of LF:** in Ado-Odo Ota, no clinical manifestation (elephantiasis or hydrocele) of LF was recorded. However 14 individuals claimed to have seen cases of swollen limbs. Seven individuals claimed to have seen cases of enlarged scrotum. All the 299 individuals examined were not aware of the cause of lymphatic filariasis and neither did they know how to treat it. A similar trend was observed in Abeokuta South where no clinical manifestation of LF (elephantiasis or hydrocele) was recorded. However five individuals claimed to have seen cases of swollen limbs while seven individuals had seen cases of enlarged scrotum. Majority of the individuals examined (93.8%) did not know the cause of lymphatic filariasis. Only five individuals knew that lymphatic filariasis was caused by mosquito bites. Other causes mentioned by the participants were: unhygienic behavior, Kwashiokor, metaphysical powers, and unhygienic water source. Swollen limbs and enlarged scrotum is known as eela and ipa respectively in the two communities.

**Entomological Survey:** in Ado-Odo Ota, a total of 56 mosquitoes (51 Anopheles gambiae s.l and 5 Culex species) were collected from different households in the community. In Abeokuta South, a total of 183 mosquitoes comprising of 122 An. gambiae s.l, 56 Culex species and 5 Aedes species were collected. None of the mosquitoes was found to be infected with microfilariae.

## Discussion

This study provided baseline indicators in two sentinel sites in Ogun State. The results show that the area is endemic for LF with an overall prevalence 4% and 2.4% in Ado-Odo Ota and Abeokuta South LGAs and are in concordance with the mapping result earlier obtained for these areas. The prevalence 4% and 2.4% reported in this study is comparatively lower than that reported by in previous studies in Ogun state [13, 14], however these earlier studies were conducted in areas with visible signs of the disease. The overall mean Mf prevalence rate that has been recorded in previous studies across Nigeria was 8.2% with a range of 0 to 47.4% [15]. The lower prevalence in Abeokuta South compared to Ado-Odo Ota could be as a result of mass ivermectin distribution for onchocerciasis treatment in the community for the past 11 years [8]. It has been shown that that long-term use of ivermectin has the ability to eliminate W. bancrofti [16]. On the hand Ado-Odo Ota is yet to commence onchocerciasis treatment.

In Ado Odo Ota, infection rates increased with age which is in conformity with what has been previously reported elsewhere in Nigeria [13, 14, 17, 18]. Seventy five percent (9 out of the 12) of the persons that tested positive in Ado Odo Ota, were between the age range of 5 to 59 years while only 3 people between the aged range 60 to 79 years were positive. The higher percentage infectivity recorded in persons older than 60 years is similar to a report obtained from the same LGA [13]. In Abeokuta south, all the 7 infected people were aged 10 to 49 years. These ages are the productive age in the communities. In Ado-Odo Ota, females had higher infection rate than males suggesting that they may be more exposed to mosquito vectors.

The microfilariae prevalence and density are the best indicators of epidemiology, management and control of LF [10]. A microfilarial density of 30.6 and 25.1 mf/ml was found in the infected population of Ado-Odo Ota and Abeokuta South LGA respectively. This microfilarial density is higher than the 22.25 mf/ml [14] and 21.4 mf/ml [13] previously reported in Ogun State. The high microfilarial density recorded in the two LGAs indicates that MDA is needed in these areas. Mean microfilarial density of 5.6 mf/50 µl [19], 9.9 mf/50 µl [20], 9.5 mfd [21] and 10.4 mfd per 20 mm3 of night blood [22] has been recorded in other parts of Nigeria.

No clinical manifestation of LF (elephantiasis or hydrocele) was recorded although some individuals claimed to have seen cases of swollen limbs and enlarged scrotum in the community. Furthermore, the individuals in the communities had local names for swollen limbs and enlarged scrotum indicating that LF was common in the communities. Majority of the individuals examined (93.8%) did not know the cause of lymphatic filariasis. Only 6.2% of people in Abeokuta South LGA knew that lymphatic filariasis was caused by mosquito bites. Other causes mentioned by the participants were: unhygienic behavior, Kwashiokor, metaphysical powers, and unhygienic water source. None of the participants from Ado-Odo Ota knew the cause of LF. However, members of both communities had seen cases of swollen limbs and enlarged scrotum. The two communities had specific local names for swollen limbs and enlarged scrotum where it is known as eela and ipa respectively.

The entomological results presented here showed that none of the mosquitoes collected was infective with microfilariae of W. bancrofti. Absence of microfilariae has been reported previously in Nigeria [23]. However, this could be as a result of the relative short period that was used for the collection. Mosquito collection over a long period of time will yield more mosquitoes and increase the likelihood of detecting the presence of microfilariae in the mosquito and for a proper conclusion to be drawn. It may also be necessary to identify W. bancrofti infection in mosquitoes by polymerase chain reaction since this method is more sensitive and cost effective than dissections [5].

The present study provides information on urban LF, and it is interesting to note a filarial prevalence of 2.4% in Abeokuta South LGA which is an urban community. There is paucity of information on urban LF transmission in Nigeria [24] and Mass Drug Administration (MDA) in Nigeria have so far been concentrated only on rural communities. This study has shown that Abeokuta south LGA and Ado-Odo Ota LGA are endemic for LF. The administering of MDA which is planned by the FMoH to commence soon will help in reducing the incidence of the infection. Vector control through the use of Long Lasting Insecticide Long Lasting Insecticidal Nets (LLINs) and other personal protection measures will help in reducing the human-vector contact.

## Conclusion

This study provides the baseline prevalence before the first round of MDA is implemented in the study area and we have ascertained that these baseline indicators will make it possible to carry out periodic evaluation of the parasitological indicators [10]. Mass awareness campaigns on the cause of LF, mode of transmission, the relationship between infection and clinical signs/symptoms and goal of MDA must be increased so as to increase acceptance and support of the control programme by the community.
